# Implications of exosomes derived from cholesterol-accumulated astrocytes in Alzheimer's disease pathology

**DOI:** 10.1242/dmm.048929

**Published:** 2021-10-26

**Authors:** Qi Wu, Leonardo Cortez, Razieh Kamali-Jamil, Valerie Sim, Holger Wille, Satyabrata Kar

**Affiliations:** 1 Department of Medicine (Neurology), University of Alberta, Edmonton, AB T6G 2G3, Canada; 2 Department of Biochemistry, Center for Prions and Protein Folding Diseases, University of Alberta, Edmonton, AB T6G 2G3, Canada

**Keywords:** Alzheimer's disease, β-amyloid, Astrocytes, Exosomes, U18666A, Cholesterol

## Abstract

Amyloid β (Aβ) peptides generated from the amyloid precursor protein (APP) play a critical role in the development of Alzheimer's disease (AD) pathology. Aβ-containing neuronal exosomes, which represent a novel form of intercellular communication, have been shown to influence the function/vulnerability of neurons in AD. Unlike neurons, the significance of exosomes derived from astrocytes remains unclear. In this study, we evaluated the significance of exosomes derived from U18666A-induced cholesterol-accumulated astrocytes in the development of AD pathology. Our results show that cholesterol accumulation decreases exosome secretion, whereas lowering cholesterol increases exosome secretion, from cultured astrocytes. Interestingly, exosomes secreted from U18666A-treated astrocytes contain higher levels of APP, APP-C-terminal fragments, soluble APP, APP secretases and Aβ_1-40_ than exosomes secreted from control astrocytes. Furthermore, we show that exosomes derived from U18666A-treated astrocytes can lead to neurodegeneration, which is attenuated by decreasing Aβ production or by neutralizing exosomal Aβ peptide with an anti-Aβ antibody. These results, taken together, suggest that exosomes derived from cholesterol-accumulated astrocytes can play an important role in trafficking APP/Aβ peptides and influencing neuronal viability in the affected regions of the AD brain.

## Abstract (precis)

**Summary:** Cholesterol accumulation within astrocytes can influence secretion of exosomes containing β-amyloid-related peptides. Uptake of these exosomes can induce neurotoxicity, suggesting their potential implication in Alzheimer's disease pathogenesis.

## INTRODUCTION

Alzheimer's disease (AD), the most common type of dementia affecting the elderly population, is characterized neuropathologically by the presence of intracellular neurofibrillary tangles, extracellular neuritic plaques, gliosis and the loss of neurons in selected brain regions (Chen and Mobley, 2019; Dawkins and Small, 2014; Lane et al., 2018). Whereas neurofibrillary tangles contain hyperphosphorylated microtubule-associated tau protein, neuritic plaques are composed of a central deposit of β-amyloid (Aβ) peptides surrounded by dystrophic neurites, activated microglia and reactive astrocytes. The Aβ peptides are generated from amyloid precursor protein (APP), which is known to be processed proteolytically by either the non-amyloidogenic α-secretase or the amyloidogenic β-secretase pathways. The α-secretase pathway is mediated by a family of disintegrin and metalloproteinase domain-containing proteins [mainly disintegrin and metalloproteinase domain-containing protein 10 (ADAM10)] that cleave APP within the Aβ domain, generating soluble APPα (sAPPα) and a C-terminal fragment (α-CTF), which is further processed by γ-secretase to produce Aβ_17-40_/Aβ_17-42_ fragments. The β-secretase pathway, on the other hand, is mediated by β-site APP-cleaving enzyme (BACE1), which cleaves APP to produce sAPPβ and an Aβ-containing β-CTF that can be processed by γ-secretase to generate full-length Aβ_1-40_/Aβ_1-42_ peptides (Andrew et al., 2016). Unlike α-/β-secretases, γ-secretase is a tetrameric complex composed of the aspartyl protease presenilin 1 or 2 (PS1/2) and three cofactors: nicastrin, presenilin enhancer 2 (PEN2; also known as PSENEN) and anterior pharynx defective 1 (APH1) protein (Andrew et al., 2016; Grimm et al., 2015). Evidence suggests that an overproduction and/or a lack of clearance may lead to increased Aβ levels, which, in turn, contribute to loss of neurons and development of AD. Although neurons are the major source of Aβ (Calhoun et al., 1999; Zhao et al., 1996), the activated astrocytes associated with plaques are also known to express APP, resulting in Aβ production (Jo et al., 2014; Nagele et al., 2003). Because astrocytes under normal conditions do not generate Aβ (Avila-Muñoz and Arias, 2014; Pihlaja et al., 2011; Thal, 2012), it is important to define the role of astrocytic Aβ in AD pathogenesis.

Astrocytes, the most abundant glial cells in the central nervous system, play vital roles in maintaining brain homeostasis, including regulation of the blood-brain barrier, trophic support, synaptic activity and synapse remodelling (Nag, 2011; Sidoryk-Wegrzynowicz et al., 2011). Upon activation, which may result from injury or development of diseases such as AD, astrocytes lose some of their normal functions and contribute to the loss of neurons (Allaman et al., 2011; Seifert et al., 2006; Steele and Robinson, 2012). A role for activated astrocytes in AD is supported by evidence that (1) they increase neuronal vulnerability to toxicity by impairing glutamate recycling (Steele and Robinson, 2012) and/or generating reactive oxygen and nitrogen species (Farfara et al., 2008; Lüth et al., 2002), (2) they express pro-inflammatory molecules such as tumor necrosis factor-α (TNF-α; also known as TNF) and interleukin-1β (IL-1β) that can increase Aβ production (Blasko et al., 2000; Li et al., 2011; Medeiros and LaFerla, 2013), and (3) they are unable to regulate efficient Aβ clearance (Mulder et al., 2012; Wyss-Coray et al., 2003) and exacerbate Aβ-mediated toxicity (Domenici et al., 2002; Garwood et al., 2011). We and others have previously reported that exposure to increasing concentration of cholesterol or sequestration of cholesterol within the endosomal-lysosomal (EL) system by treatment with U18666A, a class II amphiphile inhibiting intracellular cholesterol transport, can increase APP levels/processing, leading to enhanced Aβ production (Yang et al., 2017). Nevertheless, very little is known about the role of astrocytic Aβ in the development of AD pathology.

A number of recent studies have shown that exosomes, which are single-membrane small vesicles (30-200 nm diameter) belonging to a large family of membrane extracellular vesicles, represent a novel form of intercellular communication (Pegtel and Gould, 2019). They originate from endosomes and are secreted by most cells including neurons and glial cells (Mathieu et al., 2019; Théry et al., 2002). The exosomes, which contain a variety of proteins, lipids, glycoconjugates, mRNA, microRNA and genomic DNA, have been suggested to act as vehicles for the transfer of biomolecules/pathogens in various diseases including AD (Bellingham et al., 2012; Coleman and Hill, 2015; De Toro et al., 2015; Kalani et al., 2014; Pegtel and Gould, 2019; Simpson et al., 2008; Vingtdeux et al., 2012; Yáñez-Mó et al., 2015). The initial link with AD was established not only by the identification of Aβ in exosomes but also the presence of exosomal proteins Alg-2 interacting protein (ALIX) and flotillin-1 in Aβ-containing plaques in AD brains (Kokubo et al., 2005; Rajendran et al., 2006). Subsequent studies revealed that APP and its cleaved products are secreted with exosomes derived from neuroblastoma cell lines and primary cortical neurons (Fernandes et al., 2018; Laulagnier et al., 2018; Rajendran et al., 2006; Vingtdeux et al., 2007; Xie et al., 2019). Exosomes containing APP and its CTFs can also be taken up by other cells in which CTFs can be processed further by γ-secretase (Laulagnier et al., 2018). Inhibition of exosome secretion (Dinkins et al., 2014) or infusion of exosomes derived from cortical neurons can influence Aβ levels/deposition in mutant APP-transgenic mice (Yuyama et al., 2015), suggesting an important role for exosomes in AD pathology and its propagation. Unlike neurons, very little is known about exosomes secreted by astrocytes and their implications in AD pathology. An earlier study reported that exposure of astrocytes to Aβ triggers release of proapoptotic exosomes, which can increase cell death (Wang et al., 2012). In the present study, we show the presence of APP, APP-cleaved products (α-CTF and β-CTF), Aβ and BACE1 in exosomes derived from cultured astrocytes. Cholesterol accumulation following U18666A treatment can decrease the secretion, but enhance the levels, of APP and Aβ-related peptides in exosomes. Additionally, we show that exosomes derived from U18666A-treated astrocytes can be taken up by primary cortical neurons in a phosphatidylinositol-3-kinase (PI3K; also known as PIK3)-dependent manner and trigger cell death, suggesting an important role for astrocyte-derived exosomes in AD-related pathology.

## RESULTS

### Effects of U18666A on astrocyte-derived exosomes

**Fig. 1.**
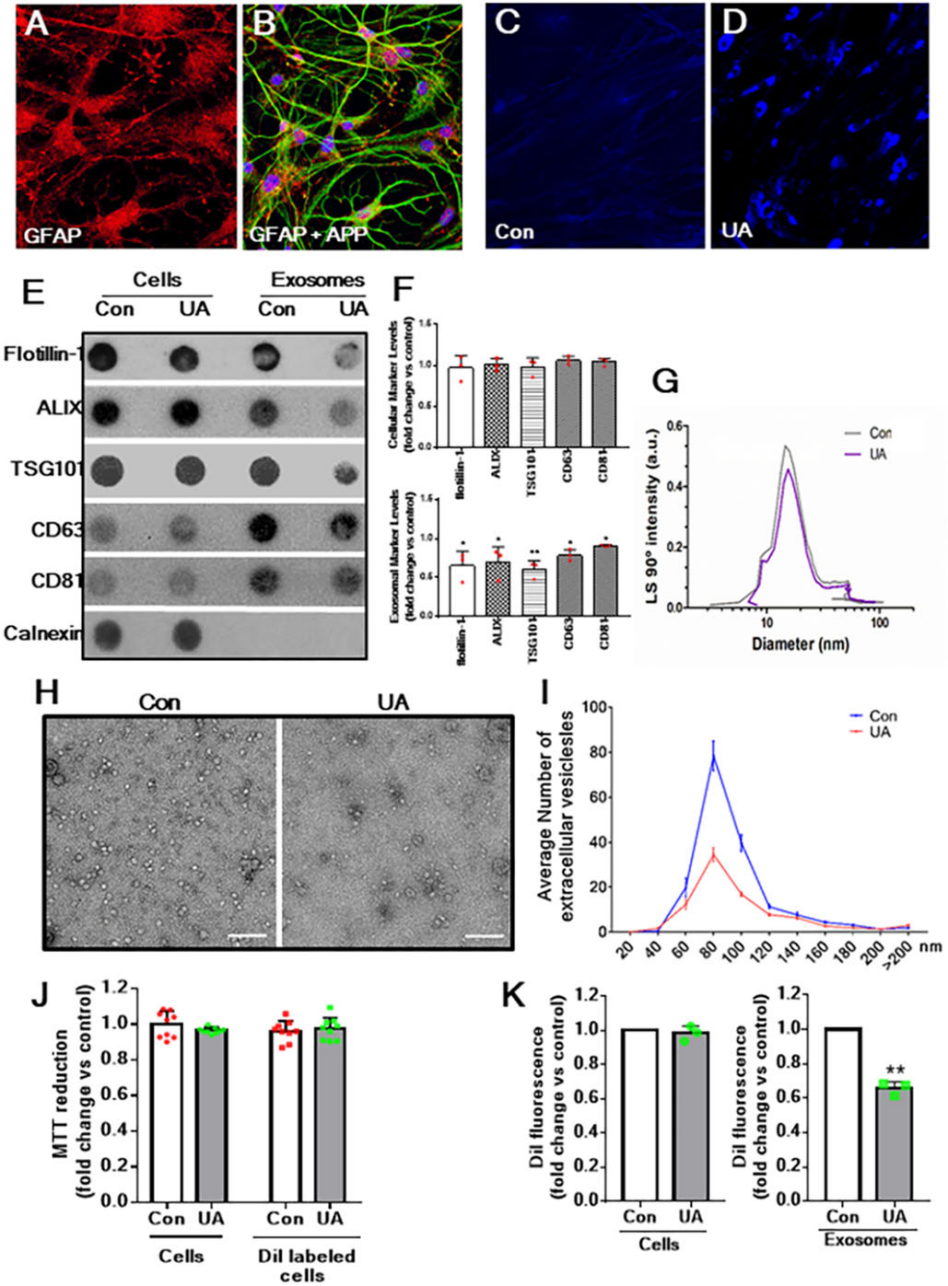
**U18666A treatment reduces exosome secretion from cultured astrocytes.** (A,B) Representative confocal images showing immunoreactive GFAP (A) and GFAP and APP (B) in control culture astrocytes. (C,D) Photomicrographs depicting the accumulation of cholesterol, as revealed by filipin staining, in cultured astrocytes treated with (D) or without (C) 5 µg/ml U18666A for 24 h. (E,F) Dot blots (E) and relative quantification (F) showing the characterization and levels of markers of exosomes isolated from control and 5 μg/ml U18666A-treated (24 h) cultured astrocytes. Note the presence of flotillin-1, ALIX, TSG101 and, to some extent, CD63 and CD81 in cells and exosomes, whereas calnexin, as expected, is evident only in the cell lysates but not in the exosomes. The levels of flotillin-1, ALIX, TSG101, CD63 and CD81 were found to be markedly decreased in exosomes isolated from U18666A-treated astrocytes compared to those from control astrocytes. (G) Dynamic light scattering (DLS) showing that the relative size of secreted exosomes did not alter between control and U18666A-treated cultured astrocytes. (H,I) Electron micrographs of exosomes isolated from control and U18666A-treated cultured astrocytes (scale bars: 200 nm) (H) and their relative size and numbers measured from 20 random electron microscopy images (I). Note the decrease in the number of exosomes secreted from U18666A-treated astrocytes compared to control astrocytes. (J) Histogram showing that neither U18666A treatment nor labelling with fluorescent dye Dil affect the viability of cultured astrocytes, as revealed by MTT assay. (K) Histogram depicting that U18666A treatment did not affect the cellular uptake of Dil fluorescent dye but decreased the secretion of Dil-labelled exosomes from U18666A-treated astrocytes compared to control astrocytes, as detected by a spectrometer. All results are presented as means±s.e.m. and obtained from three separate experiments. **P<0.01 (unpaired Student's *t*-test). a.u., arbitrary units; Con, control; UA, U18666A.

**Fig. 2.**
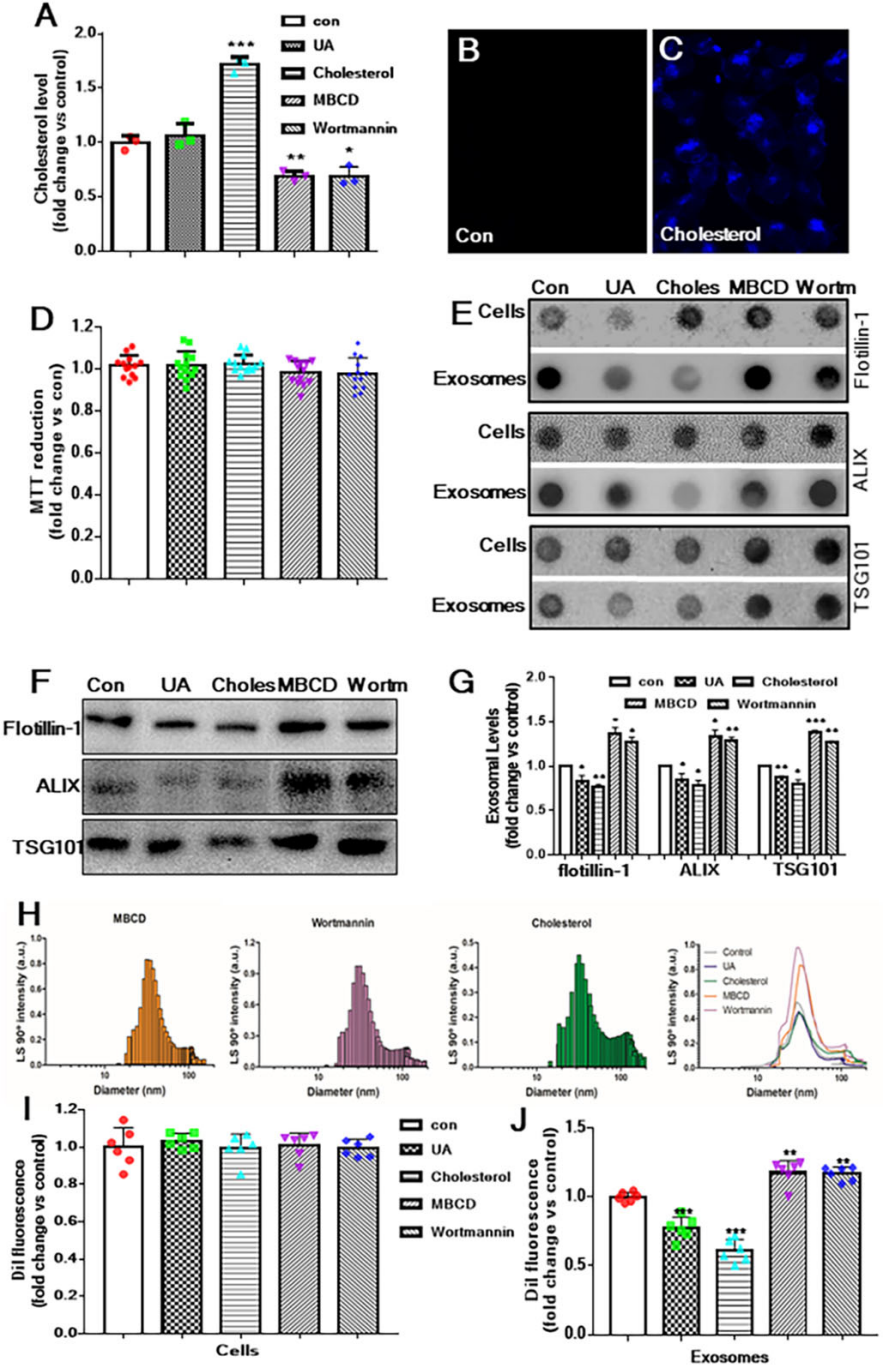
**Cholesterol level inversely regulates exosome secretion from astrocytes.** (A) Histogram showing the cholesterol levels following treatment with 5 μg/ml U18666A, 0.5 μg/ml cholesterol, 5 μM MBCD or 5 μM wortmannin for 24 h compared to control astrocytes, as measured by gas chromatography. Note that cholesterol level was not altered in U18666A-treated cultured astrocytes but increased following exposure to extracellular cholesterol and decreased after treatment with MBCD and wortmannin. (B,C) Photomicrographs depicting the accumulation of cholesterol, as revealed by filipin staining, in cultured astrocytes treated with (C) or without (B) 0.5 μg/ml cholesterol for 24 h. (D) Histogram showing that the viability of cultured astrocytes was not altered following exposure to 5 μg/ml U18666A, 0.5 μg/ml cholesterol, 5 μM MBCD or 5 μM wortmannin for 24 h, as revealed by MTT assay. (E) Dot blots showing the labelling of cell lysates and exosomes with flotillin-1, ALIX and TSG101 following treatment with 5 μg/ml U18666A, 0.5 μg/ml cholesterol, 5 μM MBCD or 5 μM wortmannin for 24 h compared to control astrocytes. (F,G) Western blots (F) and relative quantification (G) showing alterations of flotillin-1, ALIX and TSG101 in exosomal markers in cells and secreted exosomes following treatment with 5 μg/ml U18666A, 0.5 μg/ml cholesterol, 5 μM MBCD or 5 μM wortmannin for 24 h compared to control. (H) DLS showing the relative size and number of secreted exosomes following treatment with U18666A, cholesterol, MBCD or wortmannin compared to control. Note that the number of exosomes secreted decreased from U18666A- and cholesterol-treated astrocytes, but increased from MBCD- and wortmannin-treated astrocytes, compared to control astrocytes. (I) Histogram depicting that cellular uptake of Dil fluorescent dye, as measured using a spectrometer, was not altered after treatment of astrocytes with U18666A, cholesterol, MBCD or wortmannin. (J) Histogram depicting that secretion of Dil-labelled exosomes decreased from U18666A- and cholesterol-treated astrocytes but increased from MBCD- and wortmannin-treated astrocytes compared to control astrocytes. All results are presented as means±s.e.m. and obtained from three separate experiments. *P<0.05, **P<0.01 and ***P<0.001 (unpaired Student's *t*-test).

U18666A is one of the most well-characterized class-2 amphiphilic compounds to attenuate cholesterol movement from the plasma membrane to endoplasmic reticulum and from the late endosomes/lysosomes to the plasma membrane, leading to accumulation of cholesterol within the EL system (Koh and Cheung, 2006; Martin et al., 2010). As reported earlier (Yang et al., 2017), U18666A triggered sequestration of cholesterol in rat cultured astrocytes, which are characterized using the astrocyte-specific marker glial fibrillar acidic protein (GFAP) (Fig. 1A,B). In untreated cells, staining of unesterified cholesterol with filipin showed only faint labelling without any accumulation, whereas 24 h exposure to 5 µg/ml U18666A markedly increased filipin staining, suggesting intracellular sequestration of cholesterol (Fig. 1C,D). Because cholesterol sequestration enhances APP processing, leading to increased secretion of Aβ peptides (Chung et al., 2018; Yang et al., 2017), we wanted to establish whether exosomes derived from U18666A-treated astrocytes may have a role in the development of AD pathology. As a first step, we revealed that exosomes isolated from control astrocytes using polyethylene glycol (PEG)-based precipitation method (Rider et al., 2016) display established exosomal markers flotillin-1, ALIX, TSG101, CD63 and CD81 (Perez-Gonzalez et al., 2012; Raposo and Stoorvogel, 2013), but not the negative marker calnexin (Zhang et al., 2019) (Fig. 1E,F). The relative size of exosomes, as measured by dynamic light scattering (DLS), is in the range of ∼6-120 nm diameter, which is reinforced by quantification of our electron micrographs of exosomes (Fig. 1G-I). To establish whether intracellular cholesterol accumulation can influence the secretion of exosomes, astrocytes were treated with 5 µg/ml U18666A for 24 h, and then exosomes isolated from control and treated astrocytes were processed for dot-blot, DLS and electron microscopy analyses, which showed a relative decrease in the levels of markers and number, but not the size, of exosomes secreted from astrocytes (Fig. 1E-I and Fig. 2E). To validate these data, astrocytes were labelled with Dil fluorescent dye and then treated with 5 µg/ml U18666A for 24 h. Labelling of the cholesterol with Dil did not affect astrocyte viability (Fig. 1J) but decreased the secretion of exosomes from U18666A-treated astrocytes, suggesting that cholesterol accrual can decrease the amount exosomes secreted from astrocytes (Fig. 1K).

To highlight the significance of cholesterol to the secretion of exosomes, cultured astrocytes were exposed or unexposed to Dil and then treated for 24 h with various modulators of cellular cholesterol levels, such as cholesterol, methyl-β-cyclodextrin (MBCD) and wortmannin (Chung et al., 2018; Costa Verdera et al., 2017; Maulik et al., 2012; Tobert, 2003). As expected, total cholesterol levels in astrocytes detected using gas-liquid chromatography (Fig. 2A; Fig. S1A) and an Amplex Red cholesterol assay kit (Fig. S1B) were increased with cholesterol and 10% fetal bovine serum (FBS) treatment but decreased following exposure to MBCD, wortmannin and lovastatin. Cholesterol treatment, as observed with U18666A treatment, resulted in increased sequestration of intracellular cholesterol in cultured astrocytes (Fig. 2B,C). Interestingly, treatment with the aforementioned cholesterol-regulating drugs/agents did not affect the viability of cultured astrocytes (Fig. 2D; Fig. S1C). Our dot-blot and western blot analyses further revealed that cholesterol and 10% FBS treatment, as observed with UA18666A treatment, decreased the levels of exosomal markers flotillin-1, ALIX and TSG101, whereas the levels of these markers were increased following exposure to MBCD, wortmannin or lovastatin (Fig. 2E-G; Fig. S1D). This was accompanied by decreased secretion of exosomes from cholesterol- and 10% FBS-treated astrocytes, as evident from DLS analysis and/or quantification of fluorescence-labelled exosomes. By contrast, the secretion of exosomes increased following MBCD, wortmannin and lovastatin treatments (Fig. 2H-J; Fig. S1E). These results, taken together, suggest that intracellular cholesterol accumulation inversely regulates the secretion of exosomes from astrocytes.

### Effects of U18666A on exosomal APP and APP-cleaved products

**Fig. 3.**
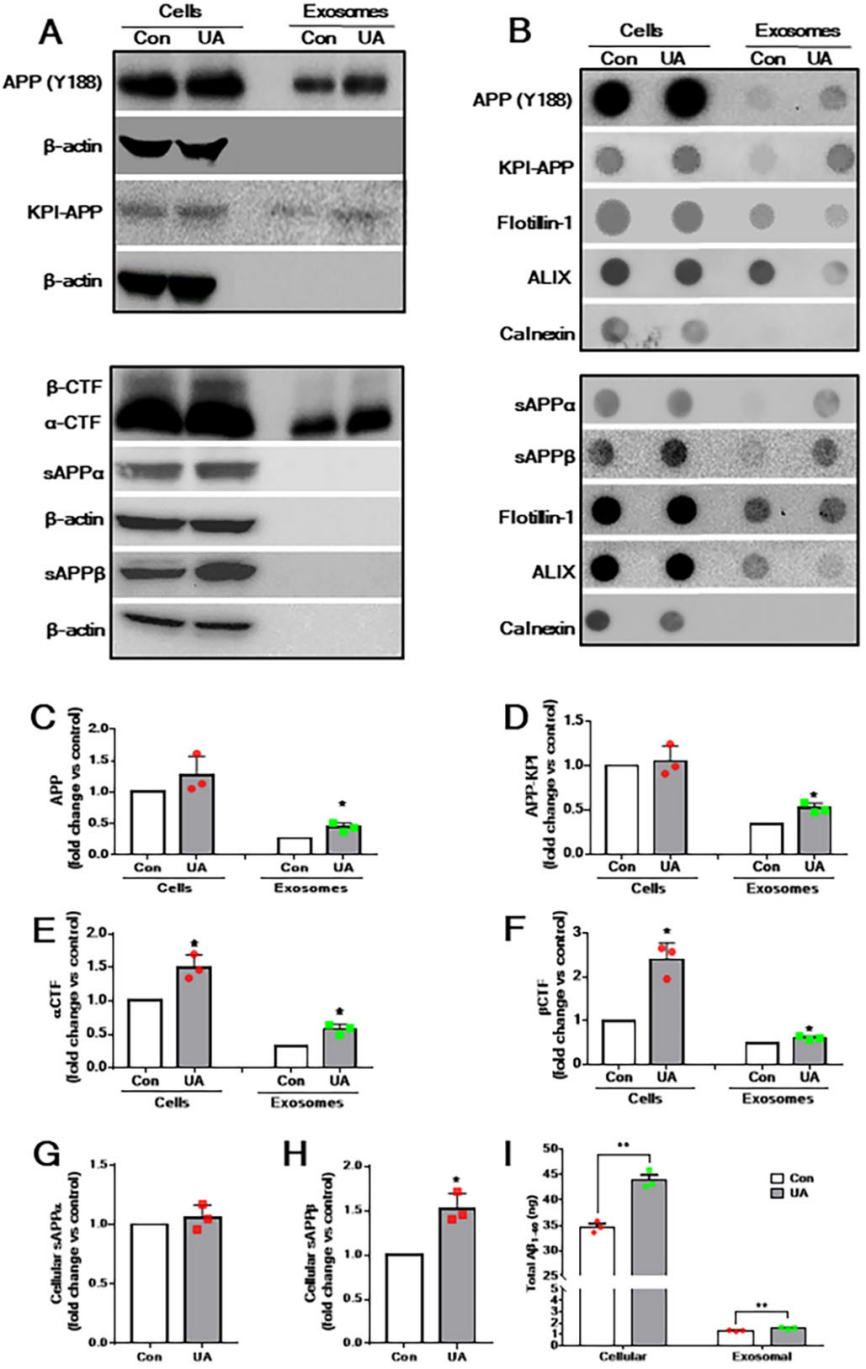
**Cellular and exosomal levels of APP and its cleaved products in U18886A-treated astrocytes.** (A) Western blots showing the levels of APP, KPI-APP, α-CTF and β-CTF in cell lysates and exosomes derived from control and U18666A-treated cultured astrocytes. Unlike in cell lysates, no sAPPα or sAPPβ was detected by western blotting in the exosomes of control and U18666A-treated astrocytes. (B) Dot blots depicting the presence of APP, KPI-APP, sAPPα and sAPPβ in cell lysates and exosomes derived from control and U18666A-treated cultured astrocytes. (C-H) Histograms showing the quantification of western blots depicting APP (C), KPI-APP (D), α-CTF (E) and β-CTF (F) in cell lysates and exosomes secreted from control and U18666A-treated astrocytes. Note the increased cellular and exosomal levels of APP, α-CTF, β-CTF and sAPPβ (H), but not sAPPα (G), following U18666A treatment compared to control. (I) Histogram depicting increased cellular and exosomal levels of Aβ_1-40_, as detected by ELISA, following U18666A treatment compared to control. All results are presented as means±s.e.m. and obtained from three separate experiments. *P<0.05 and **P<0.01 (unpaired Student' *t*-test).

Previous studies have shown that exosomes derived from cultured neurons contain APP, APP-CTFs and Aβ peptides (Fernandes et al., 2018; Laulagnier et al., 2018; Rajendran et al., 2006; Vingtdeux et al., 2007; Xie et al., 2019). However, very little is known about the occurrence of APP and its cleaved products in exosomes derived from astrocytes or its regulation by U18666A. Our western blot and dot-blot analyses revealed the presence of APP holoprotein in cell lysates and exosomes derived from control astrocytes, and its upregulation following U18666A treatment (Fig. 3A,B). This was evident not only with anti-APP antibody (clone Y188), which labels APP and APP-CTFs, but also with the antibody that identifies the Kunitz family of serine protease inhibitor (KPI)-domain containing APP, known to be expressed mostly in astrocytes (Fig. 3A,B). We also observed increased levels of APP-α-CTF and APP-β-CTF in U18666A-treated astrocytes and secreted exosomes compared to control astrocytes (Fig. 3C-F). The steady-state levels of sAPPα, but not sAPPβ, were slightly increased in cell lysates and exosomes derived from U18666A-treated astrocytes (Fig. 3G,H). Because cultured astrocytes secrete primarily Aβ_1-40_ (Yang et al., 2017), we measured the levels of rat Aβ_1-40_ using enzyme-linked immunosorbent assay (ELISA) in cell lysates and exosomes following U18666A treatment. Interestingly, the levels of Aβ_1-40_ were markedly increased in U18666A-treated astrocytes and in secreted exosomes compared to control cultures (Fig. 3I).

### Effects of U18666A on exosomal APP secretases

**Fig. 4.**
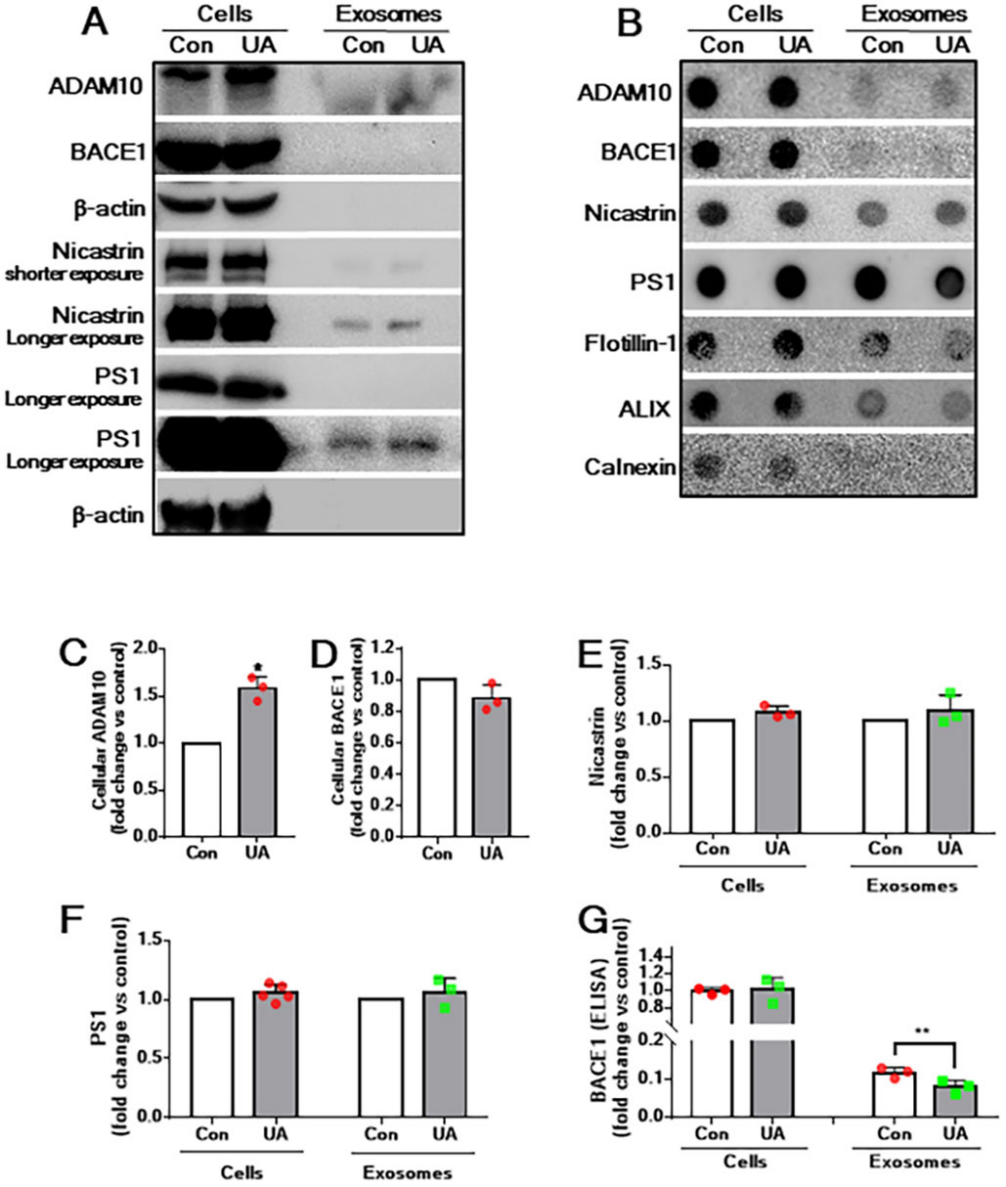
**Cellular and exosomal levels of APP secretases in U18886A-treated astrocytes.** (A,B) Western blots (A) and dot blots (B) showing the presence/levels of ADAM10, BACE1 and γ-secretase components nicastrin and PS1 in cell lysates and exosomes derived from control and U18666A-treated cultured astrocytes. (C-F) Histograms showing the quantification of western blots depicting ADAM10 (C), BACE1 (D), nicastrin (E) and PS1 (F) in cell lysates and exosomes secreted from control and U18666A-treated astrocytes. Note the increased cellular and exosomal levels of ADAM10, but not BACE1, nicastrin or PS1, following U18666A treatment compared to control. (G) Histogram depicting decreased exosomal, but not cellular, levels of BACE1, as measured by ELISA, following U18666A treatment compared to control. All results are presented as means±s.e.m. and obtained from three separate experiments. *P<0.05 and **P<0.01 (unpaired Student' *t*-test).

Earlier studies have shown that exosomes derived from cultured neurons contain APP secretases such as ADAM10, BACE1 and components of γ-secretase complex (i.e. nicastrin, presenilin, PEN2 and APH1 protein). Because α-CTF/β-CTF and sAPPα are evident in exosomes of cultured astrocytes, we evaluated the levels of ADAM10, BACE1 and two components of the γ-secretase-complex (nicastrin and PS1) in cell lysates and in exosomes derived from astrocytes using western blot as well as dot-blot analysis. Although all secretases or their components were evident in cell lysates, we were able to detect ADAM10, PS1 and nicastrin, but not BACE1, in secreted exosomes using western blot and dot-blot analyses (Fig. 4A,B). However, using a sensitive BACE1-specific ELISA, we could detect BACE1 in secreted exosomes of cultured astrocytes (Fig. 4G). Interestingly, treatment of astrocytes with U18666A, as reported earlier (Yang et al., 2017), increased the cellular levels of ADAM10, but not BACE1, PS1 or nicastrin, compared to untreated control astrocytes (Fig. 4C-F). In contrast to cell lysates, U18666A treatment did not affect the exosomal levels of PS1 or nicastrin but decreased BACE1 levels (as detected by ELISA), compared to control exosomes (Fig. 4E-G).

### Effects of U18666A on autophagy-lysosomal markers in exosomes

**Fig. 5.**
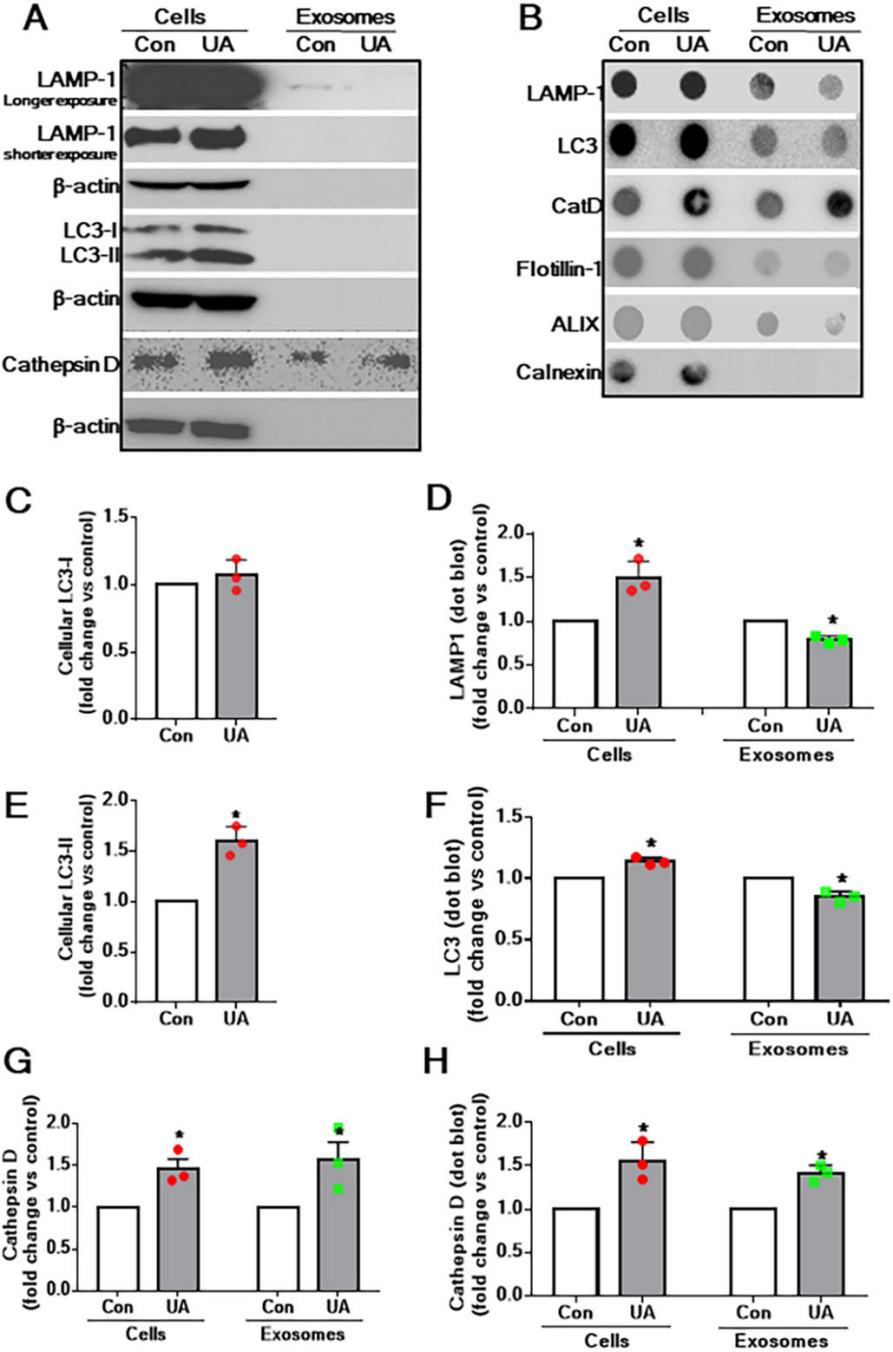
**Cellular and exosomal levels of LAMP1, LC3 and cathepsin D in U18886A-treated astrocytes.** (A) Western blots showing the levels of LAMP1, LC3 and cathepsin D in cell lysates and exosomes derived from control and U18666A-treated cultured astrocytes. Unlike in cell lysates, very low levels of LAMP1, but not LC3, were detected in the exosomes of control or U18666A-treated astrocytes by western blotting. The cellular and exosomal levels of cathepsin D were found to be increased following U18666A treatment. (B) Dot blots depicting the presence of LAMP1, LC3 and cathepsin D in cell lysates and exosomes derived from control and U18666A-treated cultured astrocytes. (C,E,G) Histograms showing the quantification of western blots depicting cellular levels of LC3-I (C), LC3-II (E) and cellular and exosomal levels of cathepsin D (G) from control and U18666A-treated astrocytes. Note the increased levels of LC3-II and cathepsin D following U18666A treatment compared to control. (D,F,H) Dot blots depicting cellular and exosomal levels of LAMP1 (D), LC3 (F) and cathepsin D (H) from control and U18666A-treated astrocytes. Note that the levels of LAMP1 and LC3 are increased in cells but decreased in exosomes, whereas cathepsin D levels are increased in both cells and exosomes following U18666A treatment compared to control. All results are presented as means±s.e.m. and obtained from three separate experiments. *P<0.05 (unpaired Student's *t*-test).

We have previously reported that U18666A treatment can enhance the levels of lysosomal [lysosomal-associated membrane protein 1 (LAMP1)] and autophagy [microtubule-associated protein 1 light chain 3 (LC3; also known as MAP1LC3B)-II] markers along with lysosomal enzyme cathepsin D in cultured astrocytes and/or neuronal cells (Amritraj et al., 2013; Yang et al., 2017). Because exosomes originate from the EL system, which plays a critical role in APP metabolism (Malm et al., 2016), we wanted to determine whether LAMP1, LC3-II and cathepsin D are evident in exosomes and altered following U18666A treatment. Our results, in keeping with the earlier studies (Amritraj et al., 2013; Yang et al., 2017), revealed an upregulation of cellular LAMP1, LC3-II and cathepsin D levels in U18666A-treated astrocytes (Fig. 5A-F). Whereas an increased level of cathepsin D was detected in exosomes in western blot and dot-blot analyses (Fig. 5G,H), a decreased level of exosomal LAMP1 and LC-3 was detected in dot-blot analyses (Fig. 5D,F).

### Exosomes from U18666A-treated astrocytes affect neuronal viability

**Fig. 6.**
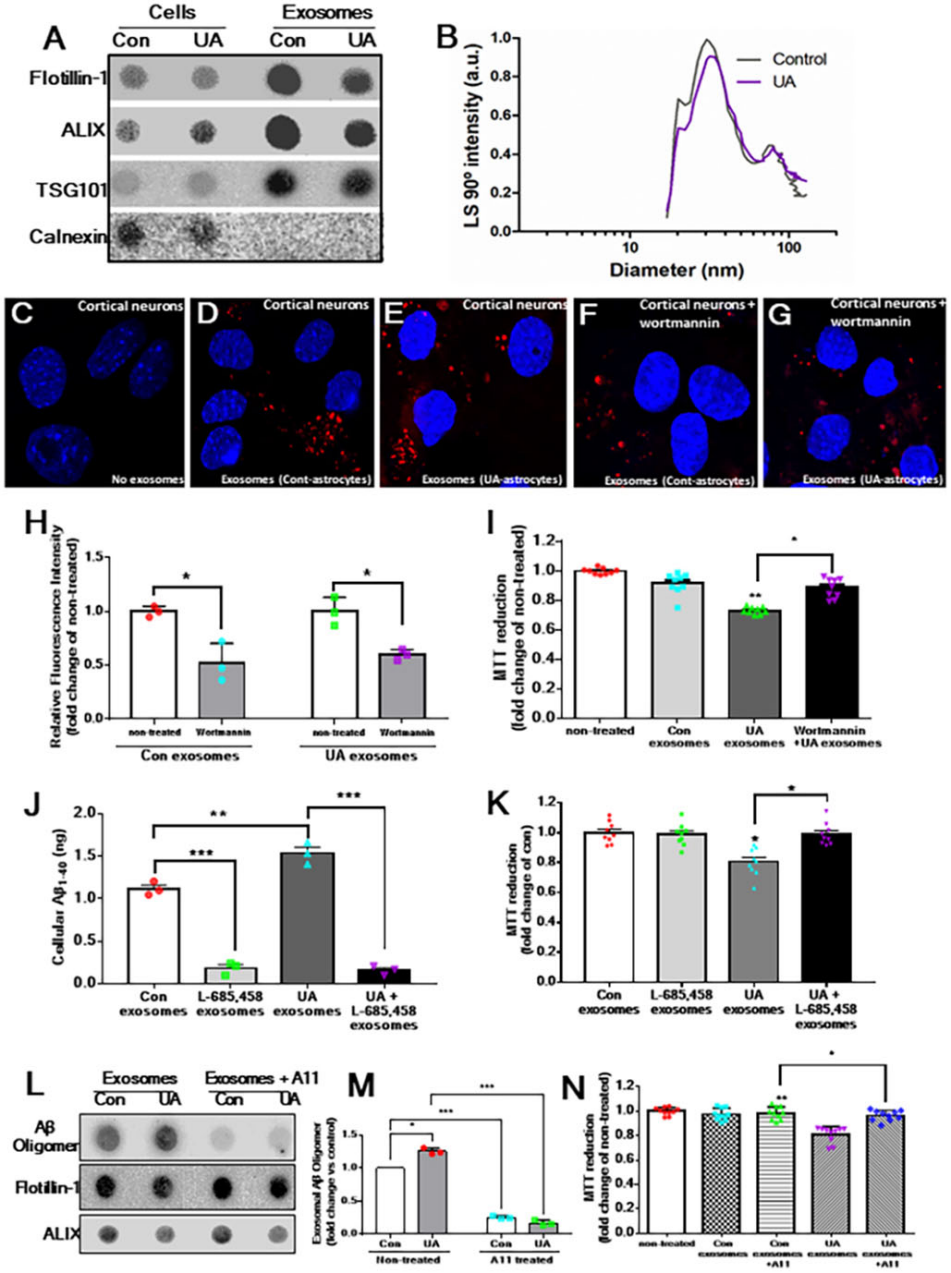
**U18666A-treated exosomes affect the viability of primary cortical neurons.** (A) Dot blots showing the presence of flotillin-1, ALIX and TSG101 in cell lysates and secreted exosomes derived from Dil-labelled control and U18666A-treated cultured astrocytes. (B) DLS showing the relative size of secreted exosomes derived from Dil-labelled control and U18666A-treated cultured astrocytes. (C-G) Photomicrographs of primary cortical neurons without exposure to exosomes (C) and following cellular uptake of exosomes from Dil-labelled control (D) and U18666A-treated (E) astrocytes in the absence (D,E) and presence (F,G) of wortmannin. Note that uptake of labelled exosomes by primary cortical neurons decreased in the presence of wortmannin. (H) Histogram showing the quantification of relative fluorescence intensity, representing decreased uptake of Dil-labelled exosomes by primary cortical neurons in the presence of wortmannin. (I) Histogram showing decreased viability of cortical neurons following uptake of exosomes derived from UA18666A-treated astrocytes compared to those from control astrocytes, as revealed by MTT assay. Inhibiting cellular uptake of exosomes from UA18666A-treated astrocytes by wortmannin protects the viability of neurons. (J) Histogram showing the cellular levels of Aβ_1-40_, as detected by ELISA, in control and UA18666A-treated astrocytes in the absence and presence of γ-secretase inhibitor L-685,458. Note that treatment of astrocytes with L-685,458 significantly decreased the cellular levels of Aβ_1-40_. (K) Histogram showing the viability of cortical neurons after uptake of exosomes derived from UA18666A-treated astrocytes treated with and without γ-secretase inhibitor L-685,458. Note the reversal of neuronal viability following uptake of exosomes derived from UA18666A-treated astrocytes exposed to L-685,458. (L,M) Dot blots (L) and quantification (M) showing the neutralization of Aβ peptide following overnight incubation of exosomes secreted from control and U18666A-treated cultured astrocytes with anti-Aβ antibody. (N) Histogram showing the viability of cortical neurons after uptake of exosomes derived from UA18666A-treated astrocytes treated with and without anti-Aβ antibody. Note the increased viability of neurons following uptake of exosomes derived from UA18666A-treated astrocytes exposed to anti-Aβ antibody. All results are presented as means±s.e.m. and obtained from three separate experiments. *P<0.05, **P<0.01 and ***P<0.001 (unpaired Student's *t*-test).

One of the critical functions of exosomes is to transport signalling and other molecules from donor to recipient cells, where they can influence their functions (Mathieu et al., 2019; Théry et al., 2002). To determine whether exosomes secreted from U18666A-treated astrocytes can affect neuronal function, we first characterized the purification of exosomes using dot-blot and DLS analyses (Fig. 6A,B) and then treated cortical neurons with 1,1′-dioctadecyl-3,3,3′,3′-tetramethylindocarbocyanine perchlorate (DiI)-labelled exosomes isolated from control or U18666A-treated astrocytes. As expected, DiI fluorescent signal was apparent only in treated cultured neurons (Fig. 6C-G), indicating uptake of exosomes by neurons. Because the PI3K pathway plays a critical role in the neuronal uptake of extracellular vesicles including exosomes (Costa Verdera et al., 2017), we pre-treated cultured neurons with the established PI3K inhibitor wortmannin and then incubated them with DiI-labelled exosomes. Interestingly, wortmannin suppressed the uptake of exosomes, suggesting that the PI3K pathway may be involved in regulating the uptake of exosomes into neurons (Fig. 6F,G). Subsequently, our results revealed that the viability of cultured neurons was decreased following exposure to exosomes derived from U18666A-treated astrocytes compared to those from control astrocytes (Fig. 6H). Interestingly, inhibiting neuronal uptake of exosomes by wortmannin attenuated the toxicity induced by U18666A-treated exosomes (Fig. 6I).

Because the level of Aβ was markedly increased in U18666A-treated exosomes, we wanted to determine the role of Aβ, if any, in neuronal vulnerability observed following exposure to U18666A-treated exosomes. Thus, we first showed that treatment with the γ-secretase inhibitor L-685,485 for 24 h markedly decreased cellular Aβ_1-40_ levels in control and U18666A-treated cultured astrocytes (Fig. 6J) without altering the characteristics or secretion of exosomes (Fig. S2A,B). Subsequently, we exposed the cultured neurons to exosomes derived from control and U18666A-treated astrocytes treated with or without L-685,485 for 24 h and then viability of neurons was assessed using 3-(4,5-dimethylthiozolyl)-2,5-diphenyltetrazolium bromide (MTT) assay. As expected, U18666A-treated exosomes reduced neuronal viability compared to exosomes derived from control or L-685,485-treated astrocytes (Fig. 6K). Interestingly, exosomes derived from U18666A+L-685,485 co-treated astrocytes significantly increased neuronal viability compared to U18666A-treated exosomes, suggesting a potential role for exosomal Aβ peptides in reducing neuronal viability (Fig. 6K). To validate this hypothesis, we subsequently evaluated neuronal viability following neutralization of exosomal Aβ with an anti-Aβ antibody (i.e. A11 antibody). Because exosomal Aβ is apparent on the surface of the exosomal membrane (Rajendran et al., 2006; Yuyama et al., 2014), incubating exosomes derived from U18666A-treated astrocytes with A11 antibody overnight neutralized exosomal Aβ, as evident from our dot-blot analysis (Fig. 6L,M). Interestingly, treatment of cortical neurons with exosomes following neutralization of Aβ peptide did not influence the cellular uptake of exosomes (Fig. S2C-F) but significantly increased neuronal viability compared to neurons treated with non-neutralized exosomes (Fig. 6N).

## DISCUSSION

The present study reveals that U18666A-induced cholesterol sequestration within the EL system can decrease the secretion of exosomes derived from cultured astrocytes but increase the levels of APP and its cleaved products in exosomes. Additionally, exosomes derived from U18666A-treated astrocytes are found to induce toxicity following cellular uptake into primary cortical neurons. This is supported by results that showed that (1) U18666A-triggered cholesterol sequestration in cultured astrocytes can decrease the secretion of exosomes, (2) levels of APP and its cleaved products APP-CTFs and soluble fragments are elevated in cells as well as in secreted exosomes following U18666A treatment, (3) cellular, as well as exosomal, Aβ_1-40_ levels are increased in U18666A-treated astrocytes, (4) exosomes derived from U18666A-treated astrocytes can be taken up by primary cortical neurons in a PI3K-dependent pathway, and (5) neuronal uptake of exosomes derived from U18666A-treated astrocytes can lead to neurotoxicity, which is attenuated by treatment of the astrocytes with a γ-secretase inhibitor or neutralization of exosomal Aβ peptide with an anti-Aβ antibody. Collectively, these results indicate that exosomes derived from cholesterol-accumulated cultured astrocytes can play an important role not only in trafficking APP and its cleaved products but also in influencing neuronal viability and spreading of AD pathology.

Evidence suggests that exosomes, generated in multivesicular bodies (MVBs), are secreted to the extracellular environment through the fusion of MBVs with plasma membrane (Colombo et al., 2014; Raposo and Stoorvogel, 2013). Cholesterol is a content of exosomes and also related closely to the process of secretion of exosomes (Pfrieger and Vitale, 2018; Tamboli et al., 2010; Xu et al., 2021). U18666A is one of the most well-characterized class-2 amphiphilic compounds, which acts not only to reduce cholesterol movement from the plasma membrane to endoplasmic reticulum and from the late endosomes/lysosomes to the plasma membrane, leading to accumulation of cholesterol within the EL system, but also to inhibit cholesterol biosynthesis by regulating enzymes involved in cholesterol biosynthesis. Additionally, U18666A affects membrane protein transport that can influence the composition of membrane and its ability to vesiculate (Cenedella, 2009; Kuzu et al., 2017). Some earlier studies reported that U18666A binds and inhibits Niemann Pick type C1 protein (NPC1), which plays a crucial role in the efflux of cholesterol out of the lysosomes, leading to its accumulation within the EL system (Liscum and Sturley, 2004; Lu et al., 2015). In the current study, we revealed that U18666A treatment can attenuate the secretion of exosomes from astrocytes but does not affect the size of the exosomes. Additionally, we showed that upregulation of cellular cholesterol level following exposure to extracellular cholesterol or 10% FBS can decrease exosome secretion, while depletion of cellular cholesterol with MBCD, wortmannin or lovastatin treatments can increase exosome secretion, suggesting an inverse role for cholesterol in regulating exosomal secretion from astrocytes. Because the transport of MVBs along the microtubule towards the plasma membrane depends partly on the cellular cholesterol content, it is possible that inhibition of cholesterol trafficking and/or synthesis by U18666A may underlie the decreased secretion of exosomes observed in treated astrocytes (Pfrieger and Vitale, 2018). Although this could be a secondary consequence of the effects of U18666A on membrane protein/structure, the evidence that upregulation and depletion of cellular cholesterol levels can inversely regulate exosome secretion/markers suggests that cellular cholesterol levels, rather than effects of U18666A (Cenedella, 2009; Kuzu et al., 2017), may be associated with the decreased secretion/markers of exosomes from cultured astrocytes. This is also consistent with a previous study that showed that U18666A can decrease the secretion of exosomes by blocking the formation of MVBs (Elgner et al., 2016). However, many studies reported differential roles of cholesterol in regulating exosome secretion, raising the possibility that variation in the levels/sites of cholesterol accumulation, cell types and/or experimental conditions may underlie the discrepancy (Chung et al., 2018; Koh and Cheung, 2006; Martin et al., 2010).

The influence of cholesterol on APP metabolism has long been studied, both in cultured conditions and in animal models of AD (Allinquant et al., 2014; Maulik et al., 2013), in view of the evidence that intracellular trafficking, localization and processing of APP are regulated by the levels of cholesterol within the cells. Consistent with earlier results, we showed that the cellular levels of APP holoproteins, including KPI-APP, known to be expressed mostly in astrocytes, are increased following U18666A treatment. In addition to APP upregulation, we observed an elevated level of α-/β-CTFs, ADAM10 and Aβ_1-40_, but not BACE1 or γ-secretase complex (PS1 and nicastrin), in U18666A-treated astrocytes. Additionally, the levels of LAMP1, LC3-II and cathepsin D, as reported in earlier studies (Amritraj et al., 2013; Yang et al., 2017), were found to increase in astrocytes following U18666A treatment. The observed changes are due to cholesterol sequestration rather than cellular degeneration, as viability of astrocytes was not found to be affected following treatment with 5 µg/ml U18666A over 24 h. Although previous studies have shown that U18666A-induced cholesterol sequestration can lead to increased levels of APP and/or its cleaved products in cultured astrocytes/neurons/cell lines (Boland et al., 2010; Chung et al., 2018; Davis, 2008; Jin et al., 2004; Koh and Cheung, 2006; Runz et al., 2002; Yamazaki et al., 2001; Yang et al., 2017), there is some discrepancy in the results, which is likely due to the cell types used in the studies along with the concentration and duration of the U18666A treatment.

Previous studies using cellular and/or animal models of AD have shown that exosomes secreted from neurons and astrocytes can serve as carriers for APP, APP-CTFs and/or Aβ peptides, which can either exacerbate or attenuate AD pathology (Fernandes et al., 2018; Laulagnier et al., 2018; Lauritzen et al., 2019; Rajendran et al., 2006; Sharples et al., 2008; Sullivan et al., 2011; Vingtdeux et al., 2007; Xie et al., 2019). However, very little is known about how different treatment strategies can regulate the levels of APP or its cleaved products in exosomes secreted from neurons or astrocytes. Our results show that U18666A-induced cholesterol sequestration in astrocytes can trigger secretion of exosomes containing higher levels of APP and APP-CTFs compared to control astrocytes. Additionally, the levels of Aβ_1-40_ are increased in exosomes derived from U18666A-treated astrocytes. It is of interest to note that, although the levels of BACE1 decrease, the levels of γ-secretase components nicastrin and PS1 remain unaltered in exosomes secreted from cholesterol-accumulated astrocytes. Because secretion of exosomes is decreased following U18666A treatment, it is likely that enhanced exosomal levels of APP and its cleaved products may have an intracellular origin due to increased cellular levels/processing of APP and their incorporation into intraluminal vesicles prior to release as exosomes. However, detection of APP and its secretases in exosomes raises the possibility that extracellular vesicles, as reported in earlier studies (Pérez-González et al., 2020), may also serve as a potential site for *de novo* APP metabolism. Consistent with our results, Aβ_1-42_ treatment, which is known to increase cholesterol accumulation within cells (Mohamed et al., 2012), has been shown to decrease exosome secretion from astrocytes in a JNK-dependent pathway (Abdullah et al., 2016). However, increased secretion of proapoptotic exosomes has also been reported from cultured astrocytes following Aβ treatment (Wang et al., 2012). Although levels of APP or its cleaved products have not been analysed in the exosomes secreted following Aβ treatment, several studies have reported the presence of APP, APP-CTFs and/or Aβ peptides in astrocytic exosomes isolated from brain or serum of mutant APP-transgenic mice (Elsherbini et al., 2020a; Lauritzen et al., 2019; Perez-Gonzalez et al., 2012, 2020). A recent study further showed that astrocyte-derived exosomes isolated from serum contain markedly higher levels of BACE1, γ-secretase, sAPPα, sAPPβ and Aβ_1-42_ than neuronal-derived exosomes in both control and AD patients, highlighting the significance of astrocytic exosomes in regulating AD pathology (Goetzl et al., 2016).

In addition to inducing high levels of APP/Aβ-related peptides, we showed that cellular uptake of exosomes secreted from U18666A-treated astrocytes can render cortical neurons vulnerable to toxicity. This effect is ameliorated by inhibiting cellular uptake of exosomes as well as by attenuating Aβ production in U18666A-treated astrocytes with a γ-secretase inhibitor, L-685,485, that did not affect exosomal characteristic/secretion, suggesting a role for exosomal Aβ-related peptides in the loss of neurons. This is further supported by the evidence that neutralization of exosomal Aβ with an anti-Aβ antibody, which did not influence neuronal uptake, was found to attenuate toxicity induced by exosomes. Although the underlying mechanisms by which Aβ triggers neuronal loss remain unclear, a recent study revealed that Aβ-containing exosomes derived from astrocytes of 5xFAD mice and AD patients can promote neurodegeneration under *in vitro* and *in vivo* conditions by inducing mitochondrial damage and caspase activation. The concentration of Aβ associated with exosomes inducing damage, however, was found to be several folds lower than those required for Aβ alone, indicating the contribution of other toxic factors in the degeneration of neurons (Elsherbini et al., 2020a,b). This is supported by an earlier study that showed that exosomes released from cultured astrocytes in response to Aβ treatment contain proapoptotic ceramide and prostate apoptosis response 4, which can trigger cell loss (Wang et al., 2012). Thus, it is of interest to determine whether proapoptotic molecules other than Aβ, such as ceramide, may have a role in the loss of neurons triggered by exosomes derived from U18666A-treated astrocytes.

Unlike neurons, astrocytes generate very little Aβ under physiological conditions due to low expression of APP and BACE1 (Thal, 2012; Zhao et al., 2011). Activated astrocytes that result from insults and pathological conditions such as AD display higher levels of APP and/or its processing enzymes, which may enhance the generation of Aβ peptides (Hartlage-Rübsamen et al., 2003; Kodam et al., 2010, 2019; Miake et al., 1999; Nadler et al., 2008; Nagele et al., 2003; Thal et al., 2000). Increased levels and/or sequestration of cholesterol within astrocytes have also been shown to enhance the production of APP and its cleaved products (Yang et al., 2017). Because cholesterol levels are increased in AD brains (Panchal et al., 2010; Xiong et al., 2008) and shown to be a risk factor for AD (Maulik et al., 2013; Wolozin, 2004), it is possible that an enhanced level/altered subcellular distribution of cholesterol within astrocytes can contribute to the development/propagation of AD pathology within the brain by triggering dysfunction/degeneration of neurons following transport of APP/Aβ-related peptides into recipient neurons. This is supported by three lines of evidence: (1) Aβ toxicity is known to be enhanced in the presence of astrocytes (Domenici et al., 2002; Thal, 2012) and following exposure to conditioned media from Aβ-treated astrocytes but not from control astrocytes (Garwood et al., 2011; Paradisi et al., 2004), (2) Aβ-containing exosomes derived from astrocytes of 5xFAD mice and AD patients can trigger neuronal loss (Elsherbini et al., 2020a,b), and (3) Aβ peptides have been shown to accumulate within astrocytes prior to plaque formation and their accrual correlates with local tissue damage in AD brains (Nagele et al., 2003; Simpson et al., 2010; Thal et al., 2000). However, contradictory data suggesting that exosomes may act as potent scavengers for Aβ in the brain (Yuyama et al., 2014) and contribute to the clearance of toxic proteins by microglia uptake (Fitzner et al., 2011; Yuyama et al., 2012) or enzymatic degradation (Katsuda et al., 2013) favour a protective role for astrocyte-derived exosomes in the development of AD pathology. Considering the multifaceted roles of different subtype astrocytes in various experimental conditions (Li et al., 2019; Liddelow and Barres, 2017), it is possible that astrocytes at early stages of AD may serve a protective role by relieving neurons from accumulated toxic materials. However, as the disease advances with compromised EL/autophagic-lysosomal system, astrocytic exosomes may act not only to propagate disease pathology but also to trigger dysfunction/degeneration of neurons in the affected brain regions (Kim et al., 2020; Upadhya et al., 2020; Venturini et al., 2019). Thus, characterizing the spatiotemporal function of exosomes derived from astrocytes from experiments relevant to disease pathology may provide a better understanding about the role of astrocytes in AD pathogenesis. In summary, the results presented in this study reveal that exosomes derived from cholesterol-accumulated astrocytes can play an important role in trafficking APP/Aβ peptides and also in influencing neuronal viability in AD brains.

## MATERIALS AND METHODS

### Reagents

Rat primary astrocytes and Astrocyte Medium-animal (AM-a) were purchased from ScienCell (Carlsbad, CA, USA), and PEG, MTT, wortmannin, cholesterol, MBCD, lovastatin, filipin and 4′,6-diamidine-2′-phenylindole dihydrochloride (DAPI) were from Sigma-Aldrich (St Louis, MO, USA). The ELISA kit for the detection of mouse Aβ_1-40_ was obtained from Life Technologies (Burlington, ON, Canada), and the ELISA kit for BACE1 was from Immuno-Biological Laboratories (Gunma, Japan). U18666A was purchased from Enzo Life Sciences (Ann Arbor, MI, USA). The radioimmunoprecipitation assay (RIPA) lysis buffer, bicinchoninic acid (BCA) protein assay kit, DiI cell-labelling solution, Amplex Red cholesterol assay kit and enhanced chemiluminescence (ECL) kit were from Thermo Fisher Scientific (Montreal, QC, Canada). Sources of all primary antibodies are listed in Table S1. Horseradish peroxidase (HRP)-conjugated secondary antibodies were purchased from Bio-Rad. All other chemicals were from Sigma-Aldrich or Thermo Fisher Scientific.

### Astrocyte cell culture

Rat primary astrocytes were cultured in AM-a containing 2% FBS, 1% penicillin/streptomycin and 1% astrocyte growth supplement-animal (AGS-a), as described earlier (Ourdev et al., 2019; Yang et al., 2017). Cells were grown in a T150 flask or 96-well plates at 37°C with 5% CO_2_, and the medium was changed every 2 or 3 days. At 90% confluence, cultured astrocytes were treated with 5 µg/ml U18666A in AM-a containing exosome-free FBS for 24 h. In some experiments, cells were treated with 5 µg/ml U18666A in the presence or absence of 100 μM γ-secretase inhibitor L-685,458, 0.5 μg/ml cholesterol, 5 μM lovastatin, 10% FBS, 5 μM MBCD or 5 μM wortmannin in AM-a for 24 h. After treatment, cells were washed, homogenized in RIPA lysis buffer containing protease inhibitor cocktail and stored at −80°C until further processing. In some experiments, exosomes isolated from control or U18666A-treated cultured astrocytes were incubated with or without anti-Aβ antibody overnight at 4°C, as reported earlier (Tang et al., 2020), and then processed for dot-blot analysis or treatment of primary cultured neurons.

### Filipin staining

Filipin labels unesterified cholesterol (Bornig and Geyer, 1974). To evaluate the intracellular cholesterol accumulation, control, U18666A-treated and cholesterol-treated cultured astrocytes were washed in phosphate-buffered saline (PBS; pH 7.4), fixed with 4% paraformaldehyde (PFA) and then incubated in the dark with 125 μg/ml filipin, as described previously (Yang et al., 2017). Stained coverslips were mounted with ProLong Gold Antifade Reagent and then visualized using an Axioskop-2 microscope (Zeiss, Oberkochen, Germany).

### Exosome isolation

Exosomes from the control and treated cultured astrocytes were isolated as described earlier (Rider et al., 2016). In brief, supernatant of the cultured astrocytes was centrifuged sequentially at 500 ***g*** for 5 min, 2000 ***g*** for 10 min at 4°C followed by 10,000 ***g*** for 30 min. Once centrifuged, the medium was added to an equal volume of 16% PEG at 4°C to achieve a desired final PEG concentration (8%). Samples were then mixed thoroughly and incubated at 4°C overnight. The next day, samples were centrifuged at 5000 ***g*** for 2 h at 4°C, the pellet was suspended in 1 ml particle-free PBS and then ultracentrifuged at 120,000 ***g*** for 90 min to wash the particles of contaminating proteins and PEG. The pellet was resuspended in 60 μl particle-free PBS by shaking for 30 min at room temperature, and the exosomes were isolated as described before (Rider et al., 2016).

### Negative stain electron microscopy

Aliquots of 5 μl exosomes derived from control and U18666A-treated cultured astrocytes were adsorbed onto freshly glow-discharged 400 mesh carbon-coated copper grids for 1 min and then washed sequentially with 50 μl 0.1 M and 0.01 M ammonium acetate solutions. The grids were then stained using a freshly filtered 2% uranyl acetate and air-dried after removing the excess stain with filter paper. The stained samples were examined with a Tecnai G20 transmission electron microscope (FEI Company, Eindhoven, The Netherlands) operating at an acceleration voltage of 200 kV. Electron micrographs were recorded with an Eagle 4k×4k CCD camera (FEI Company).

### Asymmetric flow field-flow fractionation and DLS

Asymmetric flow field-flow fractionation and DLS were used to fractionate and measure size distribution of exsosomes isolated from control and treated cultured astrocytes. Eighty microlitres of sample were injected into an AF2000 Postnova system using PBS (pH 7.4) as running buffer. The channel was 26.5 cm in length and 350 µm in height, constructed with a trapezoidal spacer of maximal width 21 mm at the inlet and lined with a 10-kDa cutoff polyethersulfone membrane at the accumulation wall. Samples were focused for 5 min and then eluted at a channel flow of 0.5 ml/min with cross-flow decreasing from 1.5 ml/min to 0.35 ml/min in the first 15 min, from 3.5 ml/min to 0 ml/min in the next 30 min, and running with no cross-flow for the last 10 min. A slot pump was run at 0.3 ml/min to concentrate the samples before they passed through the detectors. Fractions of 0.2 ml were collected, and then static and DLS measurements were carried out on an in-line DAWN HELEOS II detector (Wyatt Technology), as described previously (Eskandari-Sedighi et al., 2021; Zhang and Lyden, 2019).

### Cholesterol assay

The amount of cholesterol in the astrocytes obtained from the control and various experimental paradigms was determined using an Amplex Red cholesterol assay kit, as described previously (Maulik et al., 2012). Fluorescence was measured using a SpectraMax M5 spectrophotometer (Molecular Devices) at excitation/emission wavelengths of 560/590 nm. All samples were assayed in quadruplicate and results were from three independent experiments. The mass of cholesterol from control and experimental samples was also determined using gas-liquid chromatography. In brief, cultured samples were homogenized for 2 h at 30°C with phospholipase C and then lipids were extracted after adding internal standard Tridecanoin, before processing to determine cholesterol mass using gas-liquid chromatography, as described previously (Amritraj et al., 2009). All samples were assayed in triplicate and results were from two independent experiments.

### DiI labelling

Astrocytes suspended at a density of 1×10^6^/ml in serum-free AM-a were added to 5 μl/ml DiI cell-labelling solution, incubated for 20 min at 37°C and then centrifuged at 500 ***g*** for 5 min at room temperature, as described recently (Viveiros et al., 2021 preprint). After removing the supernatant, cells were washed with AM-a, centrifuged twice at 500 ***g*** for 5 min each and then seeded in a T150 flask with normal AM-a. The DiI fluorescence levels were measured using a SpectraMax M5 spectrophotometer at excitation/emission wavelengths of 549/565 nm.

### Dot blotting and western blotting

The protein concentrations of cell lysates collected from control and treated cultured astrocytes were determined using a BCA kit. Dot blotting was performed using the protocol of Bio-Dot Apparatus (Bio-Rad). In brief, the nitrocellulose membrane, after washing three times with 100 μl Tris-buffered saline (TBS), was spotted with 5 μl sample (containing equal amount of protein) in each well, washed with TBS, blocked with 5% milk and then incubated overnight at 4°C with various primary antibodies at the dilutions listed in Table S1. After incubation, the membranes were washed, incubated with HRP-conjugated secondary antibodies (1:5000) for 1 h, and immunoreactive proteins were detected with an ECL kit. Western blotting of cultured cells and exosomes isolated from the cells was performed as described earlier (Wu et al., 2017; Yang et al., 2017). In brief, denatured samples were resolved on 10% or 12% gradient sodium polyacrylamide gels, transferred to polyvinylidene difluoride membranes, blocked with 5% milk and incubated overnight at 4°C with various primary antibodies at the dilutions listed in Table S1. The membranes were then incubated for 1 h with HRP-conjugated secondary antibodies (1:5000), and immunoreactive proteins were detected with the ECL kit. All blots were re-probed with anti-β-actin antibody and quantified using ImageJ (Wang et al., 2020).

### Mouse cortical neuronal cultures

Timed pregnant BALB/c mice purchased from Charles River (St Constant, QC, Canada) were maintained and used according to Institutional and Canadian Council on Animal Care welfare laws, guidelines and policies. Primary cortical cultures were prepared from 18-day-old embryos of the pregnant mice, as described previously (Kodam et al., 2019). In brief, frontal cortex from pup brains was dissected in Hanks’ balanced salt solution and then digested with 0.25% trypsin-EDTA. The cell suspension was filtered through a cell strainer and plated on eight-well chamber slides or 96-well plates. The cultures were grown at 37°C in 5% CO_2_ humidified atmosphere in Neurobasal medium (Thermo Fisher Scientific) supplemented with B27/N2, 50 μM-glutamine, 15 mM HEPES, 10 units/ml penicillin, 10 mg/ml streptomycin and 1% FBS. The medium was replaced 1 day later without FBS, and all experiments were performed on day 6 or 7 after plating (Kodam et al., 2019). In brief, cortical neurons grown on eight-well chamber slides or 96-well plates were pre-treated with or without 5 μM wortmannin for 24 h and then incubated with exosomes derived from Dil-labelled control or U18666A-treated astrocytes for 4 h (for neuronal uptake) or 24 h (for neuronal viability). In parallel, neuronal uptake of exosomes treated with or without anti-Aβ antibody was also evaluated. Cells from eight-well plates were fixed in 4% PFA and processed for DAPI staining to determine exosome uptake, and neurons on 96-well plates were processed for MTT cell assay. Stained sections were examined and photographed using a Zeiss confocal microscope equipped with a 63× Plan-apochromatic oil-immersion lens (LSM 700, Zeiss).

### Cell viability assay

Viability of astrocytes, with or without DiI labelling, following various experimental paradigms, was analysed using MTT assay, as described previously (Wu et al., 2015; Yang et al., 2017). Additionally, viability of cortical neurons was evaluated following exposure to exosomes isolated from control and UA-treated astrocytes either (1) in the presence or absence of wortmannin or (2) after overnight incubation with or without anti-Aβ antibody. In another set of experiments, viability of cortical neurons was assessed following exposure of exosomes isolated from astrocytes treated with or without L-685,458 in the presence or absence of U18666A. In brief, control and treated cultures seeded on 96-well plates were replaced with new medium containing 0.5 mg/ml MTT and then incubated for 4 h at 37°C with 5% CO_2_/95% air. After the reaction, formazan was dissolved in dimethyl sulfoxide and absorbance was measured at 570 nm with a Spectramax M5 spectrophotometer. The experiments were repeated three times in triplicate.

### Immunostaining

For characterization, cultured astrocytes grown on coverslips were fixed with 4% PFA and then incubated overnight at 4°C with anti-APP in combination with anti-GFAP antibodies at the dilutions listed in Table S1. The coverslips were then exposed to Alexa Fluor 488/594-conjugated secondary antibodies (1:1000), washed and mounted with ProLong Gold anti-fade medium. Immunostained cells were visualized using a Zeiss LSM 510 confocal microscope as described earlier (Maulik et al., 2015).

### ELISA for Aβ_1-40_ and BACE1

The levels of Aβ_1-40_ and BACE1 in cell lysates and exosomes derived from astrocytes were measured using respective rat Aβ_1-40_ and BACE1 ELISA kits as reported previously (Yang et al., 2017). The OD450 value was converted to pg/ml according to a standard curve. Data represent the total Aβ_1-40_ and BACE1 levels in cell lysates and exosomes derived from cultured astrocytes. All samples were assayed in duplicate and the data were from three independent experiments (Ourdev et al., 2015; Yang et al., 2017).

### Statistical analysis

All data obtained from three to four experiments were expressed as mean±s.e.m. Statistical significance was determined by one-way ANOVA followed by unpaired Student's *t*-test for single comparison with a significance set at *P*<0.05. All analysis was performed using GraphPad Prism.

## Supplementary Material

Supplementary information
