# Supplementary material for: Implications of exosomes derived from cholesterol-accumulated astrocytes in Alzheimer's disease pathology
Source: Dis Model Mech. 2021 Oct 26;14(10):dmm048929. doi: 10.1242/dmm.048929 (PMC8560497; doi:10.1242/dmm.048929)
Supplement: Supplementary information [file dmm-14-048929-s1.pdf]

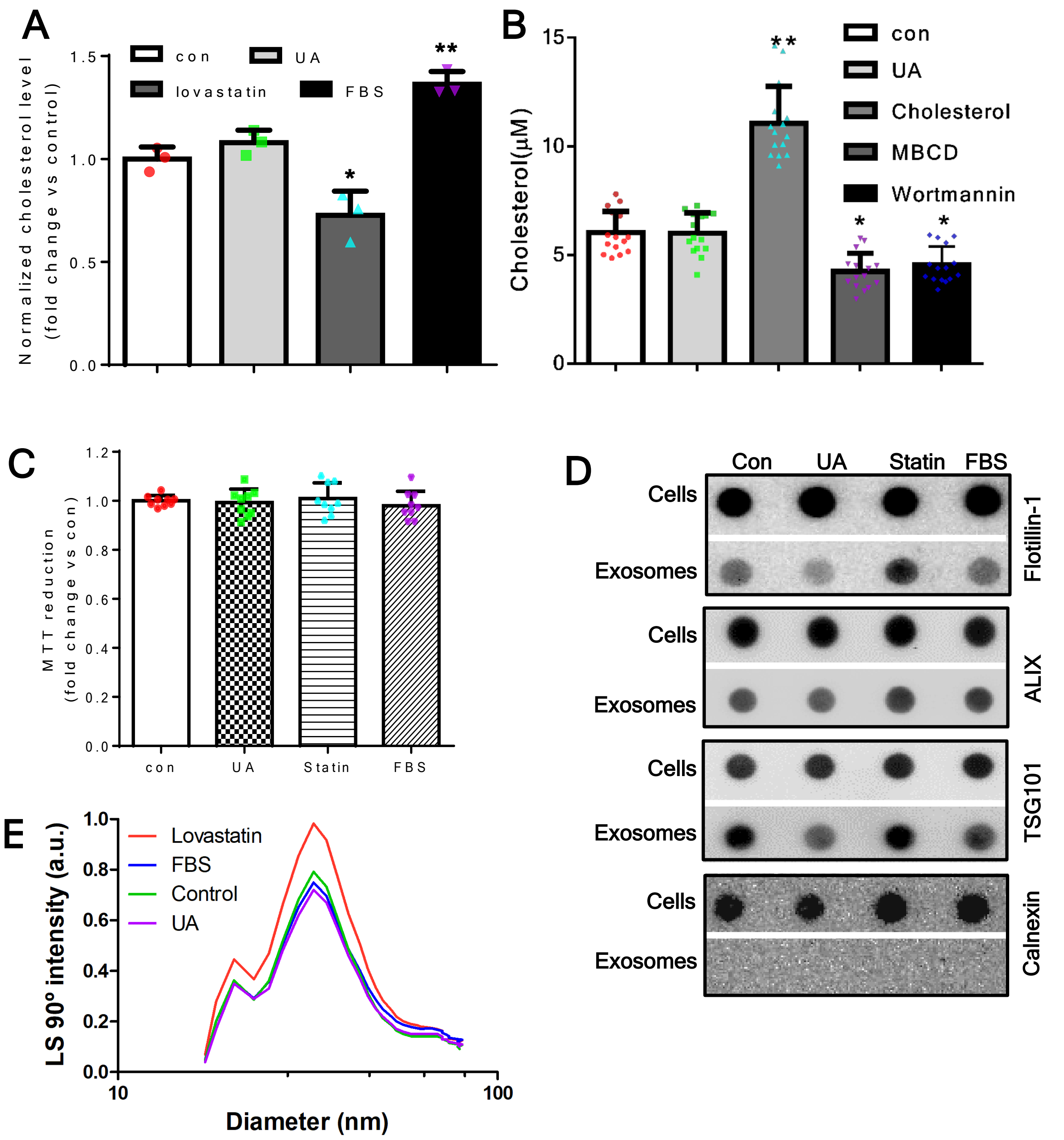

Disease Models & Mechanisms • Supplementary information

Fig. S1. Effects Lavostatin and 10%FBS on exosome secretion from astrocytes. A; Histogram showing the cellular cholesterol levels following treatment 5µg/mL U18666A, 5µM lavostatin and 10% FBS for 24hrs compared to control astrocytes as measured by gas chromatography. Note that cholesterol level was not altered in U18666A-treated cultured astrocytes but increased following exposure to 10% FBS and decreased after treatment with lavostatin. **B**; Histogram showing the cellular cholesterol levels following treatment 5µg/mL U18666A, 0.5µg/mL Cholesterol, 5µM MBCD or 5µM Wortmannin for 24hrs compared to control astrocytes. Note that cholesterol level was not altered in U18666A-treated cultured astrocytes but increased following exposure to extracellular cholesterol and decreased after treatment with MBCD and Wortmannin. **C**; Histogram showing the viability of cultured astrocytes was not altered following exposure to 5µg/mL U18666A, 5µM lavostatin and 10% FBS for 24hrs as revealed by MTT assay. **D**; Dot-blots showing the labelling of cell lysates and exosomes with Flotillin-1, ALIX and TSG101 following treatment 5µg/mL U18666A, 5µM lavostatin and 10% FBS for 24hrs compared to control astrocytes. **E**; Dynamic light scattering showing the relative size and number of secreted exosomes following treatment U18666A, lavostatin and 10% FBS compared to control astrocytes. Note that number of exosomes secreted decreased from U18666A- and 10% FBS-treated astrocytes but increased from lavostatin-treated astrocytes compared to control astrocytes. All results are presented as means ± SEM and obtained from three separate experiments. *$p < 0.05$, **$p < 0.01$ and ***$p < 0.001$. Con, control; UA, U18666A.

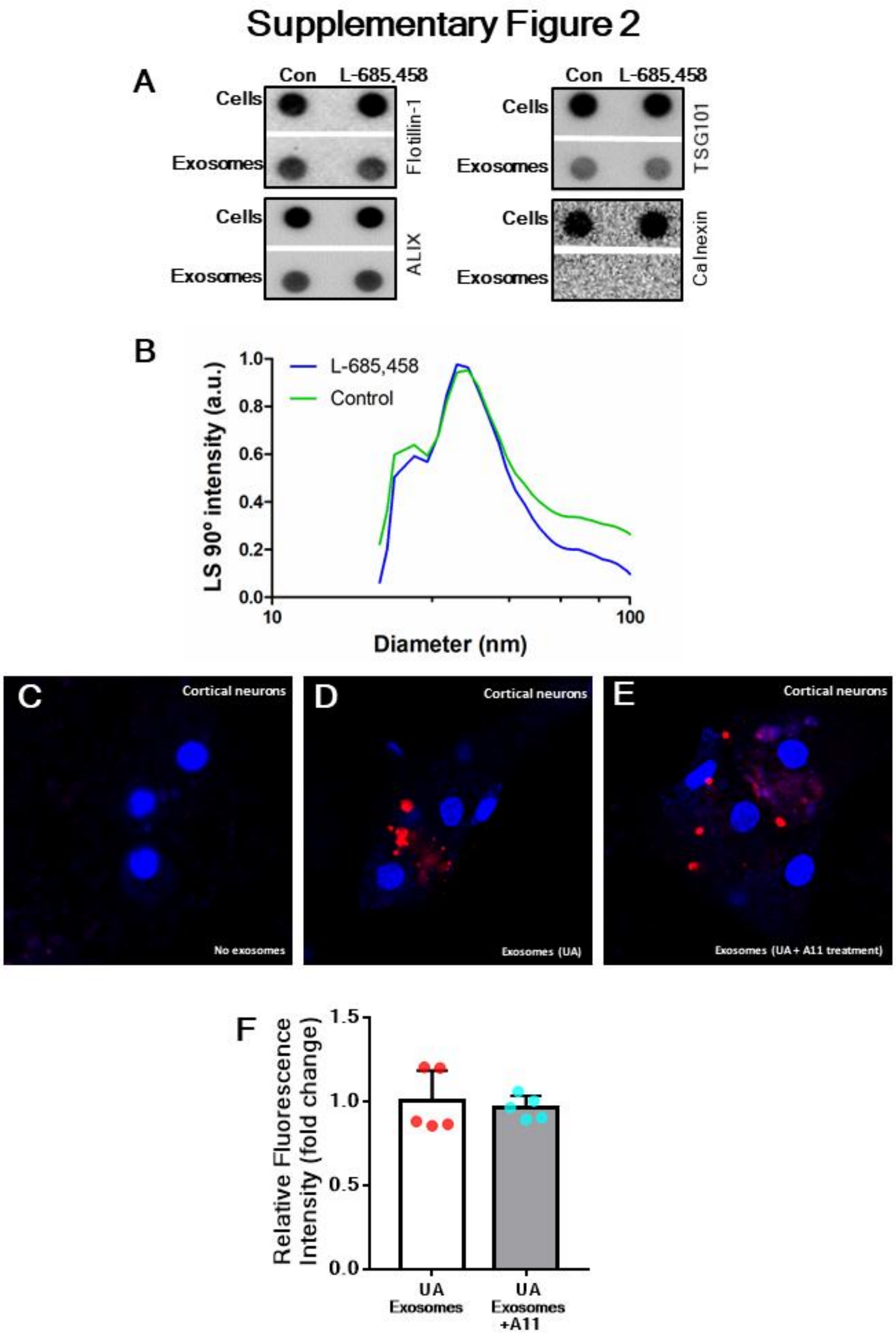

Fig. S2. Characterization of exosomes secreted following treatment with γ-secretase inhibitor and the neuronal uptake of exosomes after exposure to A11 antibody.

A; Dot-blots showing the labelling of cell lysates and secreted exosomes derived from γ-secretase inhibitor-treated cultured astrocytes in the presence or absence of U18666A with Flotillin-1, ALIX and TSG101. **B;** Dynamic light scattering showing the relative size

Disease Models & Mechanisms • Supplementary information

of secreted exosomes derived from γ-secretase inhibitor-treated astrocytes in the presence or absence of U18666A. **C-E**; Photomicrographs of primary cortical neurons without exposure to exosomes (C) and following cellular uptake of exosomes from Dil-labelled U18666A (D) and U18666A + A11-treated (E) cultured astrocytes. **F**; Histograms showing quantification of relative fluorescence intensity representing no alteration in the uptake of Dil-labelled exosomes by primary cortical neurons in the presence of A11 antibody.

Table S1. Details of the primary antibodies used in this study

Antibody type	Type	WB/DB dilution	Source
A11	Polycolonal	1:1000	Thermo Fisher Scientific
Aβ oligomers	Polyclonal	1:1000	Abcam Inc.
ADAM10	Polyclonal	1:2000	EMD Millipore Co.
APP (clone Y188)	Monoclonal	1:5000	Abcam Inc.
APP-KPI	Polyclonal	1:1000	EMD Millipore Co.
BACE1	Polyclonal	1:2000	Abcam Inc.
CD63	Monoclonal	1:1000	Abcam Inc.
CD81	Monoclonal	1:1000	Abcam Inc.
Presenilin1	Polyclonal	1:2000	EMD Millipore Co.
Nicastrin	Polyclonal	1:800	Santa Cruz Biotechnology
Soluble APPα (2B3)	Monoclonal	1:100	IBL Co., Ltd
Soluble APPβ (WT)	Monoclonal	1:100	IBL Co., Ltd
LAMP1	Polyclonal	1:1000	Abcam Inc.
LC3	Polyclonal	1:1000	MBL International Co.
β-actin	Monoclonal	1:5000	Sigma-Aldrich, Inc.
ALIX	Monoclonal	1:500	Santa Cruz Biotechnology
Flotillin-1	Polyclonal	1:1000	Thermo Fisher Scientific
TSG101	Monoclonal	1:1000	Abcam Inc.
Calnexin	Monoclonal	1:500	Santa Cruz Biotechnology

WB: Western blot; DB: dot-blot.
